# Chronic Gestational Inflammation: Transfer of Maternal Adaptation over Two Generations of Progeny

**DOI:** 10.1155/2019/9160941

**Published:** 2019-08-25

**Authors:** R. C. M. Adams, C. Smith

**Affiliations:** ^1^Department of Physiological Sciences, Science Faculty, Stellenbosch University, South Africa; ^2^Fluorescence Microscopy Unit, Central Analytical Facilities, Stellenbosch University, South Africa

## Abstract

Changes in the *in utero* environment result in generational transfer of maladapted physiology in the context of conditions such as stress, obesity, and anxiety. Given the significant contribution of noncommunicable diseases—which are characterised by chronic inflammation—to population mortality, the potential for chronic maternal inflammation mediating foetal programming is a growing concern. The extent of generational transfer in terms of immune functionality and leukocyte glucocorticoid sensitivity was investigated over two generations of offspring (F1 and F2) in a model of chronic LPS-induced maternal inflammation in C57/BL/6 mice. Maternal inflammation resulted in glucocorticoid hypersensitivity (increased glucocorticoid receptor expression levels) in the majority of leukocyte subpopulations in both F1 and F2 offspring. Furthermore, splenocytes stimulated with LPS in vitro exhibited exacerbated inflammatory cytokine responses, which were even more prominent in F2 than F1; this effect could be ascribed to NLRP3 inflammasome hyperactivity in F1 but not F2. Current data illustrates that parental chronic inflammation may mediate the inflammatory profile in offspring, potentially propagating a maladapted proinflammatory phenotype in subsequent generations.

## 1. Introduction

The high mortality resulting from noncommunicable diseases—currently accounting for over 70% of global death rates, with cardiovascular disease, cancers, diabetes, and chronic pulmonary diseases taking the forefront [1, 2]—increases the potential risk for generational transfer of maladapted physiology. This is a significant concern of modern societies, with published literature showing a precedent for transgenerational inheritance in offspring; the impact of maternal stress exposure, whether acute or chronic, is reportedly passed on to her offspring and, to an extent, her grand offspring [3–5].

The plasticity of foetal development is notoriously sensitive to environmental fluctuations, which is mediated by a variety of stressors. The foetal programming hypothesis suggests that adaptations occurring during the critical embryonic and foetal developmental stages determine the established point of physiological and metabolic responses and susceptibility to disease in later life [6]. This dysfunction is evident in transgenerational studies involving obesity [7–10], social stressors [11, 12], anxiety [13], and LPS exposure [7, 14, 15], which is, at least in part, due to changes in the *in utero* microenvironment.

The *in utero* milieu is subject to a variety of endocrine and immune adaptations, which is induced to sustain a favourable microenvironment for growth and maturation at the maternal-foetal interface. In terms of immunity, the primary alteration is the predominant type II helper T-lymphocyte (T_H_2) bias that exists during pregnancy to facilitate maternal tolerance at the maternal-foetal interface [16]. The increased progesterone, estradiol, and prostaglandin D2 (PGD2) levels during gestation seem to further encourage this T_H_2 profile, thus maintaining a relatively more immunosuppressive state in mothers [17, 18]. Maternal inflammation during gestation seems to disrupt this T_H_2 balance, resulting in a more proinflammatory T_H_1 phenotype, which adversely affects offspring. Both maternal pre- and perinatal inflammations are known to be causal in preterm birth and foetal loss [18–20]. Furthermore, it has far reaching effects on offspring behaviour [21, 22], metabolic function [7, 23], and immune functionality [7, 15, 23, 24]. Glucocorticoid and hypothalamic-pituitary-adrenal (HPA) homeostasis [22, 24, 25] is also affected by gestational inflammation. The impact of maternal stress on the foetal HPA axis in the perinatal state has been comprehensively reviewed in by Weinstock [20], who concluded that gestational stress and higher levels of maternal and foetal plasma corticosterone can result in the downregulation of foetal glucocorticoid receptors, impairing the feedback loop of the HPA axis into adulthood. Emerging evidence is suggesting that this maladaptive endocrine state may also be linked to sustained maladapted immunological functionality. For example, in rodents, stressed generation zero (F0) dams displayed higher circulating proinflammatory cytokine concentrations [15], higher leukocyte counts [7], and increased circulating cortisol levels [26]. This dysregulation was reported to persist to the F1 and F2 generations of stressed groups [12, 27, 28].

Based on previous literature in transgenerational rodent models of social stress, as well as human models of posttraumatic stress disorder (PTSD) and trauma [29–32], chronic HPA axis activation promotes glucocorticoid insensitivity, resulting in a proinflammatory phenotype, predisposing subsequent generations to increased risk of morbidity from noncommunicable disease in adulthood. Although the effects of psychological stressors during the gestational period on the maladaptation of the HPA axis have been comprehensively reported on, little data is available on the effects of chronic stressors on functional capacity of the immune response in F1 and F2 generations or their glucocorticoid sensitivity in response to chronic maternal inflammation. Thus, the purpose of our study was to delineate the plasticity of generational transfer in immune functionality and leukocyte glucocorticoid sensitivity, in a model of chronic LPS-induced maternal inflammation. Furthermore, the role of the NLRP3 inflammasome was investigated in this context.

## 2. Materials and Methods

### 2.1. Animal Experiments

**Figure 1:**
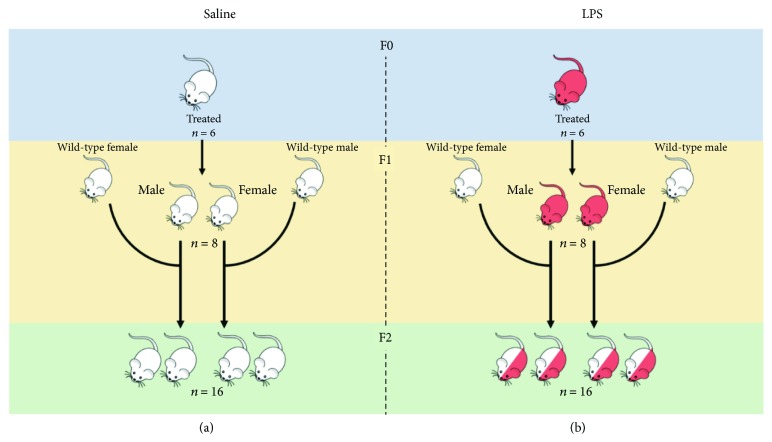
Breeding schematic for (a) saline and (b) LPS-exposed groups. The *n* represents the number of mice for each subsequent generation and gender group.

Ethical clearance was obtained from the Stellenbosch University Animal Research Ethics Committee (SU-ACUM14-00004). C57/BL/6 mice were housed under temperature-controlled conditions under a 12-hour dark-light cycle, with *ad libitum* access to standard rodent chow. After one-week acclimatization, 6-week-old dams (generation F0) were naturally mated with age-matched males. The breeding and propagation of the mice is illustrated in Figure 1. Females were placed with males overnight and removed the following morning. Successful mating was confirmed by the presence of a vaginal plug. The plug-positive dams were moved to separate cages and randomised to receive either LPS (from *Escherichia coli*; Sigma, USA; serotype 0127: B8) at 10 *μ*g/kg bodyweight, prepared in 0.9% saline solution, or 0.9% saline solution (control) at a final volume of 50 *μ*l.

The LPS or saline treatment was administered via intraperitoneal injection and repeated every seven days for the duration of gestation. The dose was based on the body mass prior to the first injection (200 ng LPS per 20 g body weight) and was kept constant throughout the duration of the gestation. A total of 3 injections were administered to each dam over the duration of gestation, which was either 19 or 20 days for all animals. For the duration of the intervention protocol, females were monitored daily for any signs of morbidity, such as lethargy, weight loss, or vaginal bleeding—none was observed. F0 was terminated 4 weeks after weaning of offspring (i.e., at 14 weeks of age and 5 weeks after the last LPS injection).

The first generation of offspring (F1), resulting from the F0 mating, was weaned at 3 weeks of age. Offspring siblings were grouped together, but sexes were separated into two cages until 8 weeks of age; after which, 4 males and 4 females per treatment group were terminated for further experimental analysis. The remaining animals were bred with wild-type animals for the second generation of offspring (F2).

For F2 generation, the F1 mice were mated with wild-type C57/BL/6 mice and the same procedure was followed for weaning but with no further intervention. Data on breeding, offspring litter size, and gestational duration did not seem to be different across generations or as result of LPS exposure (Supplementary [Supplementary-material supplementary-material-1]). As for F1, F2 mice were killed by cervical dislocation at 8 weeks of age.

### 2.2. Sample Collection

Whole blood was collected by cardiac puncture and transferred to K_2_EDTA microtubes. An aliquot was assigned for full blood and differential leukocyte counts on the CELL-DYN 3700CS haemocytometer (Abbott Diagnostics), while the remaining blood sample was used to collect plasma for assessment of corticosterone concentrations.

Corticosterone concentrations were determined by quantitative ELISA (Demeditec Corticosterone rat/mouse ELISA, Demeditec Diagnostics, Germany), as per manufacturer's instructions. The concentrations were calculated in Microsoft Excel using a 6-point standard curve with a logistic regression algorithm. The detection range of the kit was 6.1-2250 ng/ml. The kit has an intra-assay variation of 8.9% and interassay variation of 7.2%.

Mouse spleens were dissected under sterile conditions and collected into ice-cold complete RPMI 1640 medium (supplemented with 10% foetal bovine serum, 1% penicillin-streptomycin, and 1% gentamicin). Both LPS-treated dams and LPS-affected offspring displayed macroscopically visible larger spleen sizes in comparison to saline-treated dams or saline-affected offspring, respectively. Exact organ mass was however not determined due to the requirement for sterility in culturing splenocytes.

### 2.3. Cell Preparation

A single-cell suspension of murine splenocytes was generated by mechanical dissociation, by passing dissected tissue through a sterile 70 *μ*m cell strainer (BD Biosciences, USA). The cell strainer was rinsed with complete RPMI 1640 medium to remove any attached cells. Red blood cells were lysed with 1x ACK lysis buffer (150 mM NH_4_Cl, 10 mM KHCO_3_, and 0.1 mM NA_2_EDTA in ddH_2_O) for 5 minutes at room temperature, and splenocytes were washed with 1x Dulbecco's phosphate-buffered saline (DPBS, Gibco, USA). The cells were pelleted at 300×g for 5 minutes at room temperature, the supernatant was aspirated, and the pellet was resuspended in complete RPMI 1640. The cells were counted and adjusted to 1 × 10^7^/ml viable cells and used immediately for cell counting assays or frozen and stored in liquid nitrogen for subsequent batch analysis of the inflammasome and splenocyte functional capacity.

### 2.4. Basal Leukocyte Glucocorticoid Receptor Assessment

All reagents were prepared as per manufacturer's instructions prior to use. For permeabilisation of samples, the BD Cytofix/Cytoperm kit was used.

The staining buffer was prepared as 1x DPBS with 5% bovine serum albumin (Invitrogen, USA) and 1% NaN_3_ and stored at 4°C until use. The antibodies were titrated to determine optimal dilution for experiments. The antibodies and dyes and their respective dilutions are as follows: CD16/32 Fc block (BD Biosciences); Zombie Aqua Fixable Viability dye (BioLegend); NK1.1 BV421, clone PK136 (BioLegend); TCR*β* FITC, clone H57-597 (BD Biosciences); F4/80 PE-CF594, clone T45-2342 (BD Biosciences); CD11b PerCP-Cy5.5, clone M1/70 (BD Biosciences); NR3C1 Ax647, clone BugR2 (Novus Biologicals); and Ly6G APC-Cy7, clone 1A8 (BD Biosciences).

Briefly, 1 × 10^6^ splenocytes were incubated with Zombie Aqua dye in DPBS for 30 minutes at room temperature. After incubation, the cells were washed twice with DPBS and the supernatant was aspirated. The cells were then incubated with CD16/32 mouse Fc block antibody for 5 minutes in staining buffer, where after a master mix of the appropriate cell surface marker, antibodies are added. The cells were mixed thoroughly and incubated for 30 minutes at 4°C. The cells were then washed twice with staining buffer and permeabilised for 20 minutes at 4°C. After incubation, the cells were washed in 1x perm buffer and pelleted at 600×g for 5 minutes. Splenocytes were then resuspended with the appropriate dilution of the intracellular NR3C1 antibody. The samples were incubated at 4°C for 30 minutes in the dark. After incubation, the cells were washed twice with 1x perm buffer and, as a last step, resuspended in 300 *μ*l staining buffer after centrifugation. The samples were stored at 4°C for a maximum of 6 hours before acquisition using a flow cytometer.

### 2.5. Assessment of Inflammasome Activation

Splenocytes were thawed at 37°C and washed twice (300×g, 5 minutes) with prewarmed complete RPMI 1640 media. Cells were seeded at a density 2 × 10^6^/ml in 10 cm bacteriological plates in 20 ml complete RPMI 1640 media supplemented with 10% L929 media and incubated at 37°C at 5% CO_2_. On day 3, the plate was washed with prewarmed DPBS, to remove unattached cells, and the media was replaced. On day 6, the cells were harvested using 5 ml Accutase and resuspended at a concentration of 2 × 10^5^ cells per well in poly-HEMA-coated 48-well plates in 490 *μ*l RPMI 1640.

Splenocytes were incubated in RPMI 1640 medium with either LPS (100 ng/ml) (two wells per sample) or RPMI 1640 only (one well per sample) for 6 hours at 37°C. For each sample, nigericin (10 *μ*M, Sigma-Aldrich, USA) was added to one LPS well for the last 30 minutes of incubation. Following incubation, the cells were transferred into 1.5 ml microcentrifuge tubes and centrifuged at 300×g for 5 minutes to pellet cells; after which, they were fixed with 4% paraformaldehyde.

Antibodies used for labelling were titrated to determine optimal dilution for experiments. The antibodies and dyes and their respective dilutions used for the study are as follows: mouse Fc block (BD Biosciences); CD11b BV421, clone M1/70 (BD Biosciences); F4/80 PE, clone T45-2342 (BD Biosciences); pro-IL-1*β* PE-Cy7, clone NJTEN3 (eBiosciences); and ASC/TMS1 Ax647 (Novus Biologicals).

The cells were permeabilised with BD CytoFix/Cytoperm buffer for 20 minutes at 4°C and subsequently washed twice. Prior to staining, CD16/32 mouse Fc block was added to the samples for 5 minutes at 4°C to block nonspecific binding. Thereafter, a master mix of the appropriate antibodies for intracellular and extracellular markers was added and the samples were incubated for 30 minutes at 4°C in the dark. After incubation, the cells were resuspended in 1x BD perm buffer and centrifuged at 600×g for 5 minutes, at room temperature. After washing, the supernatant was discarded and the cells were resuspended in staining buffer before acquisition on the flow cytometer.

### 2.6. Flow Cytometric Acquisition and Analysis

Acquisition was performed on the BD FACSAria IIu flow cytometer (BD Biosciences), with BD FACSDiva™ version 8.1 software for data acquisition and analysis. Application settings in the BD FACSDiva software were used to standardize experimental data. As an experimental control, lot-specific 8-peak bead control was included as daily standardization validation to ensure that all settings were valid and reproducible on any flow cytometer employed for this purpose. All data files were exported as FCS 3.1 files and further analysed in FlowJo™ v10.4.2.

**Figure 2:**
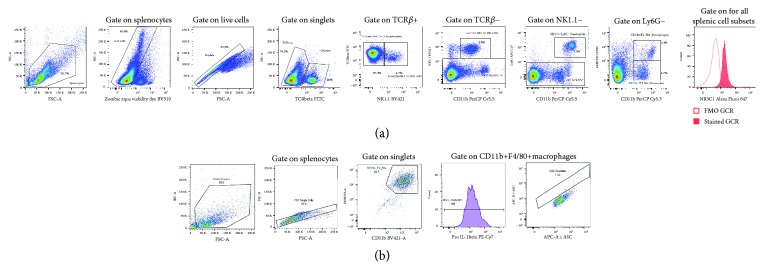
Representative images illustrating the gating strategy for basal glucocorticoid receptor expression on splenocytes (a) and assessment of inflammasome function (b).

The samples were resuspended by vortexing for 5 seconds prior to data acquisition. For the assessment of the glucocorticoid receptor expression level on specific leukocyte subpopulations, a minimum of 200 000 and a maximum of 500 000 live, gated, and singlet events were collected for each sample. The gating strategies are defined in Figure 2. Splenocytes were identified using FSC vs. SSC; thereafter, dead cells were excluded. Doublet discrimination was performed by applying a gate around the linear population in the SSC-H vs. SSC-A plot. Cells of interest were then identified from the single-cell population as follows: T-lymphocytes (TCR*β*+ NK1.1-), NKT lymphocytes (TCR*β*+ NK1.1+) NK cells (TCR*β*- CD11b+ NK1.1+), neutrophils (TCR*β*- CD11b+ Ly6G+), monocytes (TCR*β*- CD11b+ F4/80-), and macrophages (TCR*β*- CD11b+ F4/80+). Relative glucocorticoid receptor (NR3C1) expression for each cell population was quantified as relative median fluorescence intensity (MFI). Bulk gating was used to apply these gate coordinates to each generation, and all the gates were inspected and adjusted manually for each sample, if needed. All data for the experimental design was exported to Microsoft Excel.

For the inflammasome assay, a minimum of 5000 CD11b+F4/80+ macrophages were collected per sample. All samples were run on application settings, and compensation was performed every run. The gating strategies are defined in Figure 2. Macrophages were gated on the FSC vs. SSC dot plot, and doublets were excluded using FSC-H vs. FSC-A. Macrophages were further identified by CD11b+F4/80+ expression, and within this population, pro-IL-1*β* expression was quantified as relative median fluorescent intensity (MFI). Inflammasome adaptor protein Apoptosis-Associated Speck-Like Protein Containing CARD (ASC) speck formation was assessed by plotting ASC-A vs. ASC-H. The ASC speck-containing cells were gated for quantification in the doublet gate as defined by accepted methodology [33]. As a brief introduction, the inflammasome complex is a multiprotein protein structure, responsible for the tightly controlled secretion of both IL-1*β* and IL-18 that recognises pathogens via Toll-like receptor binding in combination with NOD-like receptor binding. Inflammasome assembly, and thereby the release of biologically active IL-1*β*, is a two-step process: firstly, by the production of inactive pro-IL-1*β*, stimulated by TLR ligand binding, and secondly, the formation of the inflammasome complex (ASC formation) which cleaves inactive pro-IL-1*β* into active IL-1*β* (Jha, Brickey, Pan, & Ting, 2017; Strowig, Henao-Mejia, Elinav, & Flavell, 2012). The NLRP3 is the best-studied inflammasome complex and has been implicated in obesity, heart disease, neuroinflammation, and other systemic inflammatory dysregulation (Jha et al., 2017; Menu, Vince, Vince, & Menu, 2011; Strowig et al., 2012).

### 2.7. Splenocyte *Ex Vivo* Functional Capacity

Functional capacity of splenocytes, in terms of their basal and LPS-induced cytokine secretion profile, was determined for samples from all three generations of mice. Isolated splenocytes were resuspended in RPMI 1640 at a cell concentration of 1 × 10^6^ cell/ml and plated in 24-well plates at 1 ml per well. The splenocytes were treated with either LPS (from Escherichia coli; Sigma, USA; serotype 0127: B8) at 1 *μ*g/ml in RPMI 1640 (LPS-induced/stimulated) or complete RPMI 1640 only (basal/unstimulated) and incubated for 18 hours at 37°C, 5% CO_2_. After stimulation, culture supernatants were collected and stored at -80°C for batch analysis.

The MAP Mouse Cytokine/Chemokine Magnetic Bead panel kit (Millipore, USA) was employed to assess the cytokine profile (IL-1*β*, IL-6, IL-10, TNF-*α*, and IFN-*γ*) in stimulated and unstimulated supernatant samples, using the Bio-Plex 200 system (Bio-Rad, USA) equipped with the Bio-Plex Manager™ software. Cytokine concentrations were automatically calculated based on a 6-point standard curve (in duplicate) fitted with a five-parameter logistic regression algorithm. The lowest limit for the detection of IL-1*β*, IL-6, TNF-*α*, IL-10, and IFN-*γ* was 1.1 pg/ml, 2.3 pg/ml, 2.0 pg/ml, and 1.1 pg/ml, respectively. The highest limit of detection was 10 000 pg/ml for all cytokine kits used.

### 2.8. Data Reduction and Statistical Analysis

For flow cytometric data, percentage of cells for each leukocyte population identified and median fluorescent intensity (MFI) were used in statistical analyses. All data was exported to Microsoft Excel from respective analysis programs and consolidated. Data was analysed in Statistica version 13.2 (StatSoft Software, USA), and graphs were generated in GraphPad Prism 7.04 (GraphPad Software Incorporated, USA). After confirming normalcy of data distribution, one-way analysis of variance (ANOVA) was performed for the F0 LPS and saline comparison and a two-way analysis of variance (ANOVA) was employed for the F1 and F2 comparison. Fisher's LSD post hoc tests were employed to analyse the statistical significance of differences between control and LPS-affected groups within the same generation. Data is presented as means and standard errors of the mean (SEM), and *p* < 0.05 or less was regarded as significant.

## 3. Results

### 3.1. Gestational Chronic LPS-Induced Inflammation Affects Maternal Physiology Even after the Recovery Period

After 3 weeks of recovery, plasma corticosterone levels were similar between control and LPS-exposed groups. Insufficient numbers per group exclude firm conclusions on this, but individual data (Supplementary [Supplementary-material supplementary-material-1]) seems to suggest that at this time point, the majority—but not all—mothers have fully recovered in terms of circulating glucocorticoid levels.

As mentioned above, spleen size for LPS-treated dams and their offspring was visibly increased, in line with reports in other models of inflammation [34, 35]. Blood and splenocyte absolute counts and relative distributions are summarized in Table 1. In line with the glucocorticoid (GC) data, in circulation, neither total nor differential leukocyte counts differed between groups. When considering splenocyte counts, the same representation is seen, with the exception of splenic eosinophil counts, which were significantly higher (*p* < 0.05) in the LPS-exposed group. It is possible that activated neutrophils were counted as eosinophils by the automated cell counter. Since it was not logistically possible to exclude this possibility through manual assessment of blood smears, this result was excluded from interpretation. (Given the relatively low eosinophil counts, it is unlikely that neutrophil counts would have been significantly affected.)

**Figure 3:**
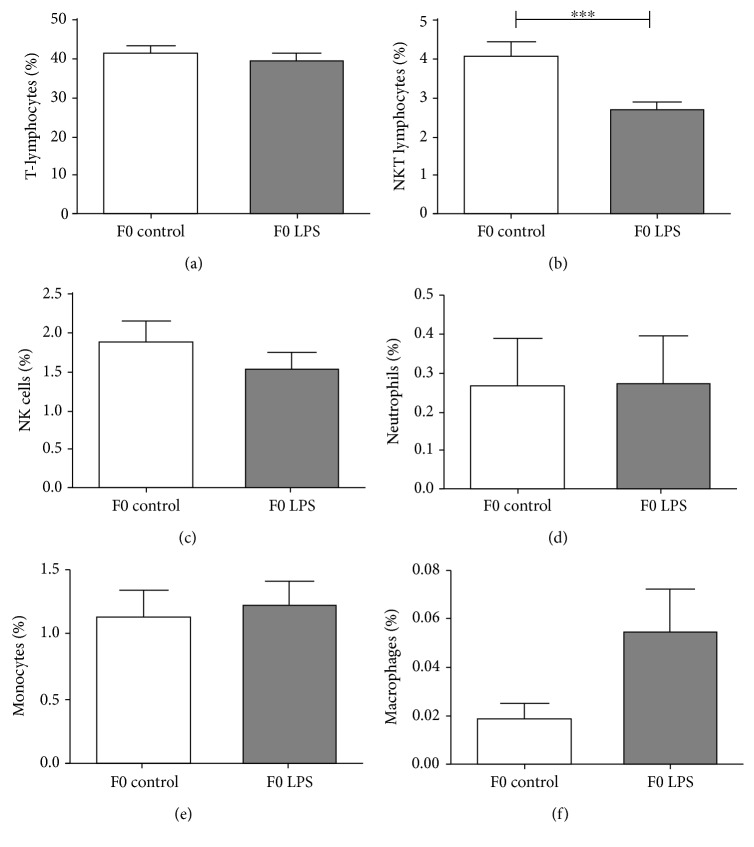
Frequency distribution of splenocyte populations between control and LPS-exposed female mice (F0), 3 weeks after the final LPS challenge: (a) T-lymphocytes, (b) NKT-lymphocytes, (c) NK cells, (d) neutrophils, (e), monocytes, and (f) macrophages. One-way ANOVA was used to compare F0 LPS and F0 saline groups. Data is represented mean ± SEM. F0 control, *n* = 5; F0 LPS, *n* = 6. Significance is depicted as follows: ${}^{***}\underset{¯}{p}<0.001$.

When analysing relative distribution of splenocytes using a flow cytometer (Figure 3), again, most cell types appeared to have returned to control levels, except for NKT-lymphocytes, which remained relatively lower in the LPS-exposed group even after 5 weeks of recovery.

**Figure 4:**
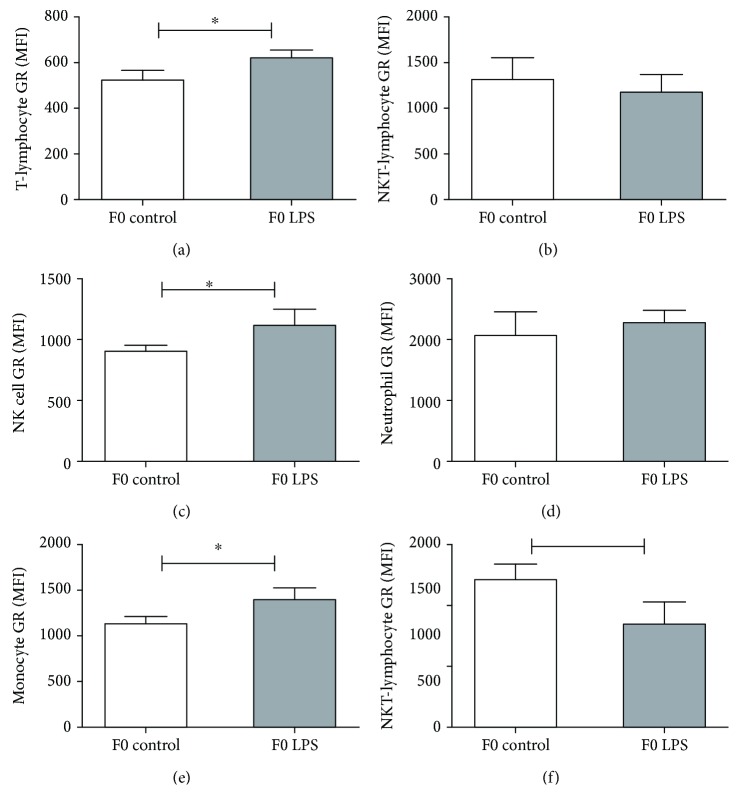
Basal cytoplasmic glucocorticoid receptor protein expression levels on splenocyte populations collected from F0 control mice vs. mice 3 weeks after repeated LPS treatment: (a) T-lymphocytes, (b) NKT-lymphocytes, (c) NK cells, (d) neutrophils, (e) monocytes, and (f) and macrophages. One-way ANOVA was used to compare F0 LPS and F0 saline groups. Data is represented mean ± SEM. F0 control, *n* = 5; F0 LPS, *n* = 6. Significance is depicted as follows: ^∗^*p* < 0.05 and ^∗∗^*p* < 0.01.

Relative basal GR expression in response to repeated LPS exposure in F0 mice differed between different types of splenocytes at a time point 3 weeks after the last LPS challenge (Figure 4). Previous LPS exposure resulted in maintained relatively higher GR expression on T-lymphocytes, NK cells, and monocytes but lower GR expression on macrophages and seemingly no lasting effect on neutrophils and NKT-lymphocytes.

Both basal and LPS-induced capacities of splenocytes to secrete proinflammatory cytokines were not significantly affected by previous repeated LPS exposure (Supplementary [Supplementary-material supplementary-material-1]). However, a general pattern seems to exist for both pro- and anti-inflammatory cytokine levels, which were on average slightly higher in previously LPS-exposed groups. This of course remains to be substantiated in a larger group of animals.

### 3.2. Transgenerational Inheritance of Chronic LPS Exposure

Plasma corticosterone levels for the F1 and F2 generations showed no significant response to the F0 maternal intervention, when comparing LPS groups to their respective generational controls, albeit somewhat inconclusive due to limited sample size (Supplementary [Supplementary-material supplementary-material-1]).

**Figure 5:**
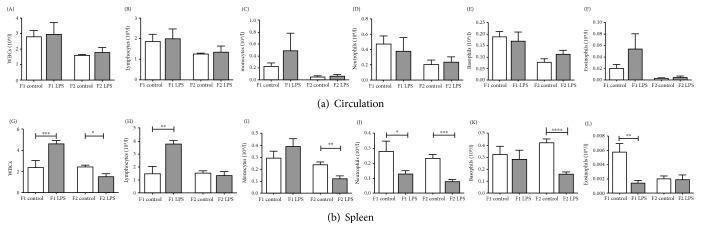
Total and differential leukocyte counts in peripheral blood circulation (A–F), and spleen (G–L) from LPS-affected vs. control mice across two generations of offspring from LPS-treated F0 mothers. Two-way ANOVA and Fisher's post hoc tests were used to compare LPS and saline groups. Data is represented mean ± SEM, *n* = 8 per group. Significance is depicted as follows: ^∗^*p* < 0.05; ^∗∗^*p* < 0.01; ^∗∗∗^*p* < 0.001, and ^∗∗∗∗^*p* < 0.001.

In terms of leukocyte counts (Figure 5), total and differential leukocyte count was unaffected in circulation (Figure 5, A–F). In contrast, total count was significantly increased in spleens of generation 1 LPS-affected (F1_LPS_) mice but significantly decreased in F2 LPS-affected (F2_LPS_) animals (Figure 5, G–L). Total lymphocyte counts showed significantly higher in association with inherited LPS exposure in both generations. Interestingly, counts for both neutrophils and basophils—the early phase proinflammatory role players—were significantly lower in spleens of LPS-affected animals, an effect that was even more pronounced in F2 than F1. A similar effect was also seen for splenic monocytes, with statistical significance only reached in F2.

**Figure 6:**
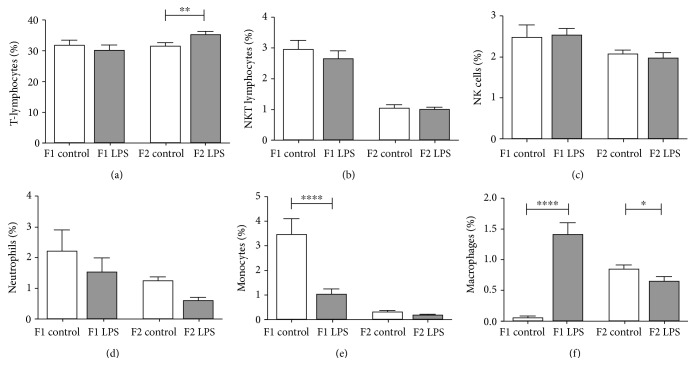
Frequency of splenocyte populations between LPS-affected and control F1 and F2 generations. (a) T-lymphocytes, (b) NKT-lymphocytes, (c) NK cells, (d) neutrophils, (e), monocytes, and (f) macrophages. Two-way ANOVA and Fisher's post hoc tests were used to compare LPS and saline groups. Data is represented mean ± SEM, *n* = 8 per group. Significance is depicted as follows: ^∗^*p* < 0.05; ^∗∗^*p* < 0.01; ^∗∗∗^ < 0.001, and ^∗∗∗∗^*p* < 0.001.

The relative frequencies of specific splenocytes mirrored absolute count data, also showing lower relative counts for neutrophils and monocytes in response to ancestral LPS exposure (basophils were not assessed) (Figure 6). Although relative counts for macrophages again reflect a decrease in association with LPS for F2, the opposite is seen in F1—this may indicate a phenotype switch to favour F4/80+ macrophages. In addition, this analysis revealed that the increased splenic lymphocyte count can be ascribed to T-lymphocytes rather than NKT-lymphocytes or NK cells, which appeared unaffected by the LPS intervention.

**Figure 7:**
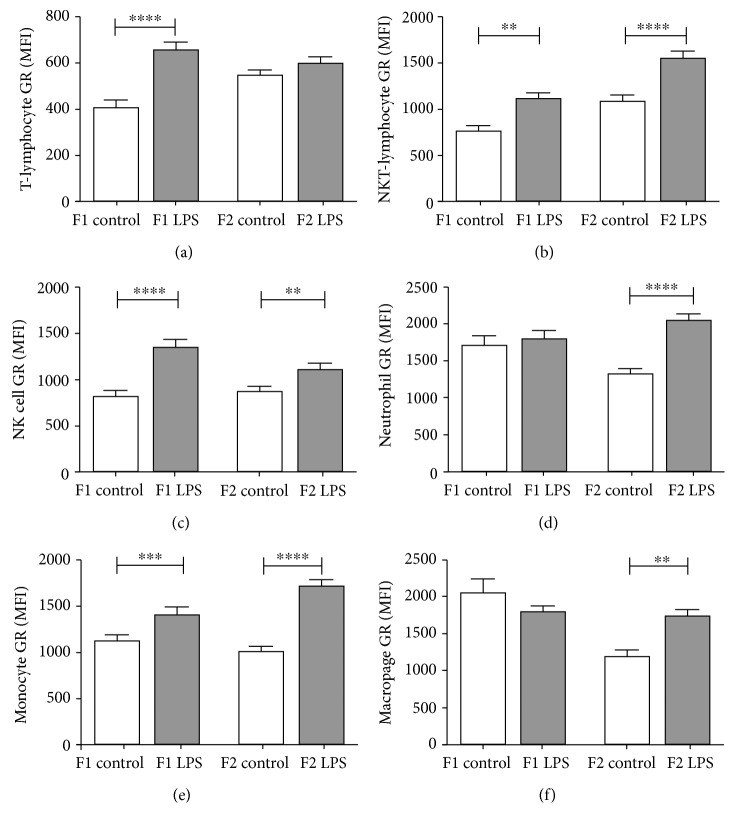
Glucocorticoid receptor levels in spleen T-lymphocytes (a), NKT-lymphocytes (b), NK cells (c), neutrophils, (d) monocytes, (e) and macrophages (f) of F1 and F2-untreated and LPS-treated generations. Two-way ANOVA and Fisher's post hoc tests were used to compare LPS and saline groups. Data is represented mean ± SEM, *n* = 8 per group. Significance is depicted as follows: ^∗∗^*p* < 0.01; ^∗∗∗^*p* < 0.001, and ^∗∗∗∗^*p* < 0.001.

Turning attention to GR expression levels in offspring, the majority of leukocyte types exhibited increased GR expression levels in response to the LPS intervention (Figure 7). Of interest, two exceptions were evident—for F1, both neutrophil GR and macrophage GR seemed unaffected. However, when considering F0, F1, and F2 responses to LPS together, this generation seems to reflect a transitional phase to effects only statistically and significantly evident in F2.

In terms of functional capacity, basal cytokine secretion was not affected by ancestral LPS exposure in either F1 or F2, with levels mostly below kit detection thresholds (Supplementary [Supplementary-material supplementary-material-1]).

**Figure 8:**
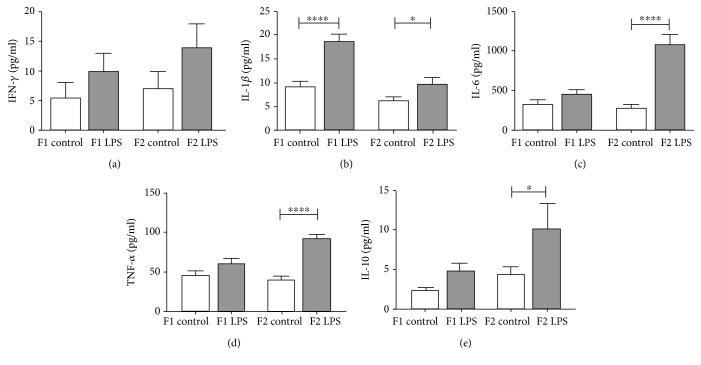
LPS-stimulated *ex vivo* cytokine responses of splenocytes from control vs. LPS-affected mice across two generations of offspring. IFN-*γ* (a), IL-1*β* (b), IL-6 (c), TNF-*α* (d), and IL-10 (e) levels were analysed after 18-hour incubation with 1 *μ*g/ml LPS. Two-way ANOVA and Fisher's post hoc tests were used to compare LPS and saline groups. Data is represented mean ± SEM, *n* = 8 per group. Significance is depicted as follows: ^∗^*p* < 0.05; ^∗∗^*p* < 0.01; ^∗∗∗^*p* < 0.001, and ^∗∗∗∗^*p* < 0.001.

However, in response to *in vitro* LPS challenge, the nonsignificant increase in cytokine production seen in mothers even after a period of recovery (Supplementary [Supplementary-material supplementary-material-1]), which suggests a transient cytokine response to the LPS stimuli, was propagated and seemed to become more significant with each subsequent generation for the majority of cytokines assessed (Figure 8).

### 3.3. Contribution of NLRP3 Inflammasome

**Figure 9:**
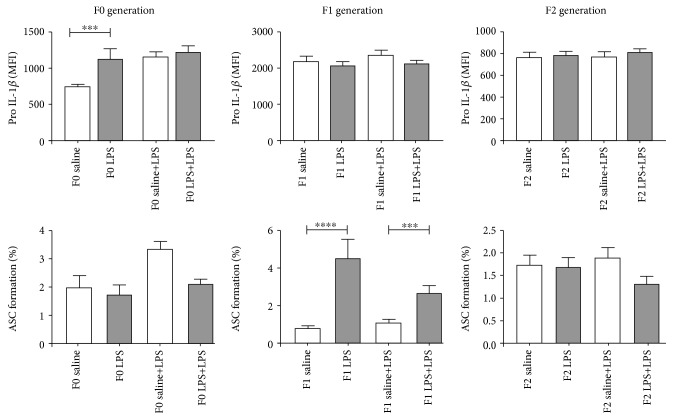
Relative expression levels of Pro-IL-1*β* and ASC formation in CD11b+F4/80+ splenic macrophages. Basal and LPS responses were assessed in F0, F1, and F2 for both LPS and control groups. Two-way ANOVA and Fisher's post hoc tests were used to compare LPS and saline groups. Data is represented mean ± SEM, *n* = 8 per group. Significance is depicted as follows: ^∗∗∗^*p* < 0.001 and ^∗∗∗∗^*p* < 0.001.

In the current study, mouse splenocytes were frozen for an extended period of time (approximately 6 months) to facilitate batch analysis of all generations. In these previously frozen cells, the addition of nigericin proved to be unnecessary to facilitate conversion to IL-1*β*. Thus, only basal and LPS-induced inflammasome activation is presented (Figure 9). The control F0 splenic F4/80+ macrophages exhibited the expected LPS-induced increase in pro-IL-1*β* production, as well as conversion to IL-1*β* (as indicated by ASC complex formation). In the LPS-exposed group, basal intracellular pro-IL-1*β* levels were significantly higher (*p* < 0.05) when compared to controls. However, relatively less efficient conversion occurred in response to acute LPS challenge. In generation F1, cells seem to maintain constant pro-IL-1*β* expression levels but, *in utero* LPS exposure, was associated with more efficient conversion to IL-1*β* both basally and in response to acute LPS challenge. In F2, no significant differences are evident between control and LPS-exposed offspring.

## 4. Discussion

The current study has successfully established an *in vivo* mouse model of chronic maternal inflammation, by expansion of the maternal periconception systemic inflammation (MPSI) protocol established by Williams et al. [15]. For the current model, the low-dose (10 *μ*g/kg, every 7 days) LPS intraperitoneal administration in pregnant dams was continued until the end of the gestation period. Significant alterations in the immunological and HPA functionality are reported for all generations.

Chronic MPSI induced postnatal changes in the HPA axis, as well as in the leukocyte profile and functional capacity in mothers; some of which remained evident even after a recovery period. Furthermore, significant lasting effects of the LPS-affected *in utero* microenvironment are evident in the F1 and F2 generations, with regard to both circulating and reservoir immune cells, as well as glucocorticoid responsiveness and cytokine responses to *ex vivo* LPS challenge.

The current data contributes to the knowledge regarding transgenerational inheritance of physiological adaptations to gestational chronic inflammation. The detailed leukocyte subpopulation-specific analyses, in particular, provide new insight into the role of an altered *in utero* environment on the immunological phenotypes of both the F1 and F2 generations of offspring.

### 4.1. Maternal Adaptation to Gestational Chronic LPS Administration

In terms of gestation and litter size, neither was significantly affected by chronic MPSI, although offspring number was lower than that reported after either single-dose LPS [15] or immobilisation stress during gestation [36], suggesting that the current model could represent a comparatively more severe stressor.

An acute single low-dose (10 *μ*g/kg) LPS challenge is known to induce an inflammatory cytokine response in dams, which return to control levels by 72 hours postadministration [15]. Similarly, in a mild model of chronic systemic administration (6.45 *μ*g/kg LPS per day, administered by osmotic pump during pregnancy and lactation) [7], LPS-administered dams displayed no significant impact on the postnatal systemic inflammatory profile at one week after cessation of LPS challenge. However, in the current study, a relatively more severe LPS challenge (weekly bolus injection of 10 *μ*g/kg) resulted in a long-term reduction in splenic NKT lymphocyte counts. In addition, in line with published data on GR expression generated in total leukocytes or total lymphocytes in circulation [37], macrophage GR was indeed lower in response to the chronic inflammatory stimulus. However, in contrast, subpopulation-specific analysis indicated an increased GR expression on T-lymphocytes, NK cells, and monocytes—minority cell types which may not necessarily reflect in total T or total leukocyte GR analyses—suggesting a more differential leukocyte response to this particular chronic stress than previously thought.

The gestational LPS challenge indicated more significant effects on NKT cell numbers in the spleen, which is in line with and expand on available relevant literature. The low splenic NKT-lymphocyte frequencies reported here are likely due to NKT cell migration from the spleen into the maternal decidua [38, 39]. Alternatively, a decreased availability of NKT cells for storage in the spleen may result from increased recruitment of NKT cells from circulation into the maternal decidua. Previously, LPS exposure and risk of foetal loss were attributed to the increased presence of invariant NKT lymphocytes (iNKTs) in the maternal decidua [40]. iNKTs contribute to the majority of NKT numbers and typically coexpress T-lymphocyte receptors as well as NK cell receptors. iNKTs are significantly implicated in LPS-induced pregnancy loss [41] and preterm delivery [19, 39, 41] through inflammatory cell activation as well as T_H_-1 and T_H_-17 responses. This interpretation is in line with findings in a pilot study to the current study, where the initiation of LPS-induced inflammation prior to conception prevented pregnancy in the majority of dams (data not shown). Furthermore, the NKT adaptation in the current study was sustained for several weeks after the end of the administration of the LPS stimulus, suggesting an inability of pregnant dams to readily recover, which may have predisposed their offspring to related (mal)adaptations.

Assessment of GR expression levels in a leukocyte subpopulation-specific manner provided more information. Clinical evidence has substantiated the hypothesis that chronic stress directly influences leukocyte GR expression and functionality, although some contrasting data has been reported. For example, in PTSD veterans, lower total leukocyte GR density was reported [42], specifically in T-lymphocytes, B-lymphocytes, and NK cells [43]. In contrast, PTSD and anxiety disorders have also been associated with elevated lymphocyte GR expression [44], specifically in neutrophils [45]. This is possibly the result of glucocorticoid adaptation occurring across a continuum where GR levels initially increase in response to acutely increased glucocorticoid levels, followed by GR downregulation when glucocorticoid hypersecretion becomes chronic. In terms of inflammation, this results firstly in an anti-inflammatory effect, which changes to a more proinflammatory outcome with the onset of glucocorticoid resistance. In the current study, significantly higher GR expression was evident in T-lymphocytes and NK cells in response to maternal chronic gestational LPS exposure. The higher GR levels in T-lymphocytes may, at least in part, be due to the presence of T-regulatory cells, which promote autoimmune protection during pregnancy [46]. Future detailed analysis could shed more light on the validity of this interpretation. Moreover, even under normal conditions, NK cells are particularly sensitive to glucocorticoids [47]. It is thus not unexpected that this cell type in particular would respond by further increasing GR and thus GC sensitivity under conditions of chronic inflammatory activation. This may further indicate that the current protocol was not long enough in duration to result in chronic downregulation of GC sensitivity, i.e., the dams did not have a sustained proinflammatory phenotype. However, given the continuum of GR adaptation, if offspring were to inherit a hyperresponsiveness to glucocorticoids—as seen here in NK cells and T-lymphocytes—they may be at risk of reaching the threshold for glucocorticoid insensitivity relatively earlier in life. This is in line with the earlier incidence of noncommunicable diseases in the modern era [48–50].

Monocyte and macrophage populations and their response to LPS are well characterised. In the current study, monocytes indeed exhibited an increased GR expression in dams exposed to LPS, which is in line with the relevant literatures [37, 51, 52] and an interpretation of a predominating inflammatory profile existing in the LPS-treated dams. In contrast, macrophages had decreased GR expression levels in response to LPS. Similar to current data, splenic macrophages previously displayed insensitivity to glucocorticoids and enhanced IL-6 production in a model of chronic social stress [53]. Although the corticosterone levels were not indicative of glucocorticoid insensitivity in the current study, the modulation of splenic cell composition and GR levels, which was also reported in another model of chronic mild stress [45], suggests a selective GR insensitivity in the splenic macrophages, priming the immune system to a relatively more proinflammatory phenotype.

Elucidation of splenic macrophage NLRP3 activation in the chronic LPS MPSI model, through the expression of pro-IL-1*β* and ASC, further supports this interpretation. In the current study, when compared to saline-exposed controls, chronic LPS administration resulted in higher unstimulated pro-IL-1*β* production by F4/80+ CD11b+ splenic macrophages of F0 dams. This finding corresponds to the increased basal pro-IL-1*β* production-reported macrophages of aged mice [54], as well as in chronic LPS exposure in rats, where significantly higher IL-1*β* secretion is reported at the basal level [55]. Interestingly, when an acute added stressor is applied, IL-1*β* secretion is unaltered from the basal level [55], which is again in line with the current data. This suggests that the NLRP3 complex formation has a rate-limiting step in its response to chronic stress, possibly to prevent continuous activation. This is supported by the literature, which names caspase-1 as a rate-limiting enzyme in the cleavage of pro-IL-1*β* to release IL-1*β*, in the context of neuroinflammation [56].

More knowledge is undoubtedly required to fully elucidate all mechanisms and role players involved, particularly within a human model. Nevertheless, gestational exposure to chronic inflammation clearly results in significant effects on the mother which is not readily resolved and may thus be transferred to her progeny as a relatively proinflammatory phenotype already at birth.

### 4.2. Transgenerational Inheritance of Chronic LPS Administration in F1 and F2 Generations

There is a growing body of evidence in support of generational transfer of increased susceptibility to disease and dysregulation. For example, in diabetes and obesity, there is a significant association between parental history of obesity and diabetes and levels of serum fatty acid-binding protein 4, retinol-binding protein 4, and adiponectin, favouring obesity the risk developing of these disorders in offspring [57]. Furthermore, the foetal-maternal uterine environment is reported to have direct effects on offspring weight and metabolic outcomes [58]. Diet-induced obesity has also been associated with altered splenic CD4+ cells, macrophages, and dendritic cells in mice [59] and elevated inflammatory cytokines and risk of mortality [60]; with this, metabolic dysfunction is also seen to be transferable to offspring in murine studies [61]. In the current study, both F1 and F2 generations of offspring to the LPS dams exhibited significantly compromised physiology. Our data is in line with the abovementioned findings and also expands on the current knowledge, illustrating that this inherited “proinflammatory proneness” may not be limited to specific diseases but could be more generally applicable to any parental chronic exposure involving substantial inflammation.

In line with previous reports [15, 62], maternal LPS exposure did not affect offspring basal corticosterone levels when compared to controls. This is in support of our interpretation of inherited inflammatory proneness, as F1_LPS_ exhibited HPA axis hyperactivation, while F2_LPS_ displayed that an HPA axis response may be attributed to relative adrenal burnout [63]. This may also explain the rise in GR in the majority of splenocytes assessed for F1. However, F2_LPS_ splenocytes maintained the increased GR levels when compared to F2 control mice, perhaps as a countermeasure to the relative adrenal hyporesponsiveness. Thus, in response to chronic LPS exposure, maladaptation in offspring may be indicated by changes in HPA functionality rather than GR levels specifically.

In our opinion, the GC and GR hyperresponse in F1_LPS_ splenocytes reported is due to an inflammatory phenotype resulting from maternal inflammatory response and subsequent placental inflammatory responses, which was shown to induce indirect foetal damage, specifically intestinal damage, which persists way beyond the postnatal period [64].

In the current study, the placental inflammatory response may explain the changes in leukocyte numbers in F1_LPS_. While circulating leukocyte number and distribution remained unaffected, a clear picture of an inherited altered immune system is evident in splenocytes. *In utero* inflammatory response to maternal LPS exposure was associated with increased splenocyte counts, primarily due to a rise in lymphocyte populations. In contrast, neutrophil numbers were relatively decreased—which we interpret as tissue sequestration of neutrophils participating in affected areas of inflammation. This is further supported by flow cytometry results indicating an increased conversion of splenic CD11b+ monocytes to CD11b+F4/80+ macrophages that primarily produce IL-1*β*. Although the proinflammatory phenotype observed in F1_LPS_ had no increased capacity for NLRP3 activation or increased proinflammatory cytokine secretion basally, during acute LPS challenge, both NLRP3 conversion of pro-IL-1*β* to active IL-1*β* and proinflammatory cytokine secretion were significantly exacerbated in F1_LPS_ in comparison to controls. Chronic preconditioning with inflammatory mediators, such as IL-1*β*, has been shown to induce immunotolerance, by downregulation of TLR4 and through stimulation of corticosterone production [65], which may be the case in F1_LPS_.

When considering F2_LPS_, a picture of relative immune tolerance seems to emerge, suggesting that effects of chronically elevated IL-1*β* on TLR4 in F1 may have been inherited by F2_LPS_. For example, in contrast to the increase in splenic WBCs in F1_LPS_, in F2_LPS_, splenic WBCs were decreased. Most notably, macrophage frequency decreased in F2_LPS_. In addition, the available macrophages failed to activate NLRP3 in response to stimulus, effectively resulting in a much smaller net IL-1*β* response upon acute *in vitro* challenge, in line with TLR4 downregulation. This picture of relative immune tolerance was associated with an approximate (but statistically insignificant) 20% decrease in GC levels.

Interestingly, the sustained upregulation of leukocyte GR into the second generation of offspring may suggest epigenetic adaptation or primordial germline inheritance adaptation. This is also in line with a previous report with upregulated GR in response to chronic mild psychological stress [45]. In the study by Nephew and colleagues, the hypomethylation of GR gene promotor regions was lost in the second-generation offspring. However, their model was milder than the one employed in the current study [28]. A review by Dunn, et al. [8] hypothesised that for a phenotype to be inherited into one subsequent generation, *in utero* modification (epigenetic adaptation) is the only requirement. However, for a phenotype to be inherited into two or more subsequent generations, the stimulus has to be robust for resulting adaptations to stably alter germ cells. Taken together, the current study and that of Nephew et al. [28] suggest that the plasticity of generational transfer may be severity-dependent. Importantly, it also indicates that this adaptation is not limited to infectious stimuli.

The increased leukocyte GR expression showed increased secretion of IFN-*γ*, TNF-*α*, IL-6, and IL-10 in F2_LPS_, a response not significantly present in F1_LPS_. Previously, increased induced TNF-*α* [12] and IL-6 [66, 67] were also reported in a model of chronic social stress, again suggesting a stimulus-independent adaptive effect. Furthermore, data suggest independence of this adaptation from the NLRP3 inflammasome. Nevertheless, the increased levels of cytokines other than IL-1*β* suggest an alternative source, possibly lymphoid cells or neutrophils.

Though basal cytokine levels do not allude to a systemic proinflammatory phenotype, which have been reported in other stress models [12, 28, 68], there are two potential mechanisms that we propose to this outcome. Firstly, in F1_LPS_, the upregulation of glucocorticoid sensitivity possibly reduced cytolytic activity by reduction of histone promoter acetylation for perforin and granzyme B and TNF-*α*, IL-6, and IFN-*γ* production, as previously reported for NK cells [47, 69]. However, in F2_LPS_, where a picture of relative GC hyposecretion seemed present, this downregulation may have been abolished, resulting in increased cytokine release from NK and potentially also other cells. Secondly, neutrophils specifically are negatively implicated in the primordial generational programming in the F2 generation, as acute high-dose LPS have been shown to increase neutrophil counts as well as the GR receptor level on neutrophils [70] and GR expression on macrophages [71]. Furthermore, impaired translocation of activated GR was also demonstrated in at least neutrophils and T-lymphocytes after LPS exposure [70]. Thus, in a chronic stimulus setting, sustained impaired GR function on specific leukocytes is probable and in line with current results suggesting a relative proinflammatory outcome despite downregulated macrophage NLRP3 and increased GR expression levels. Future purpose designed studies could shed further light on this probability.

When specifically considering the leukocyte GR data, the response seen in the F0 model is mirrored in F1 and F2, with exacerbation of the response with each successive generation. This expands on the generally accepted report ascribing reduced hippocampal GR promotor methylation in offspring from low grooming arch back nursing (LG-ABN) mothers to diminished GR sensitivity, which persisted to adulthood [5]. Our current data is also in line with data from a transgenerational chronic social stress (CSS) mouse model, where lower GR methylation was reported in F1 CSS offspring but not in F2 CSS [28]. This may allude to epigenetic inheritance or primordial germline inheritance, due to the chronic grandparent and *in utero* parental exposure to LPS, with even higher responsiveness in the F2 generation despite the addition of an unaffected parent.

## 5. Conclusion

In the current study, we show that in a mouse model of chronic low-grade maternal inflammation induced by LPS administration, long-standing reprogramming of the offspring phenotype may occur and this effect was perpetuated in the next generation without any further stimulus. The maternal inflammatory state is mirrored and exacerbated in two subsequent generations of offspring, suggesting transgenerational inheritance of the inflammatory phenotype and perhaps underlying epigenetic adaptation. The current results also suggest that similar primordial germline programming may occur in response to LPS and/or psychological stressors. Thus, the current study contributes to our understanding of parental contribution to predisposition for the development of noncommunicable chronic diseases.

Given these insights, it is imperative to confirm and further characterise the plasticity and mechanisms underlying this “proinflammatory programming,” particularly in a human model. Furthermore, potential sex-dependent differences should be investigated.

## Figures and Tables

**Table 1:** Peripheral blood and splenic differential leukocyte counts in the F0 control and F0 LPS-exposed groups. One-way ANOVA was used to compare F0 LPS and F0 saline groups. Data is represented as means ± SD.

	Compartment	F0 control (1 × 10^9^ cells/L)	F0 LPS (1 × 10^9^ cells/L)
Total leukocytes	Circulation	2.82 ± 0.41	3.15 ± 0.70
	Spleen	3.80 ± 0.54	4.04 ± 0.55

Lymphocytes	Circulation	1.29 ± 0.48	1.37 ± 0.33
	Spleen	3.11 ± 0.48	3.24 ± 0.69

Monocytes	Circulation	0.147 ± 0.061	0.094 ± 0.048
	Spleen	0.231 ± 0.037	0.279 ± 0.074

Neutrophils	Circulation	1.21 ± 0.78	1.57 ± 1.05
	Spleen	0.127 ± 0.041	0.256 ± 0.129

Eosinophils	Circulation	0.024 ± 0.013	0.020 ± 0.007
	Spleen	0.016 ± 0.004^a^	0.032 ± 0.016^b^

Basophils	Circulation	0.152 ± 0.092	0.090 ± 0.059
	Spleen	0.319 ± 0.063	0.382 ± 0.115

LPS: lipopolysaccharide. Results are depicted as mean ± SEM. F0 control, *n* = 5; F0 LPS, *n* = 6. Significance: ^ab^*p* < 0.05.

## Data Availability

The data used to support the findings of this study are available from the corresponding author upon request.

## Abbreviations

ASC: Apoptosis-Associated Speck-Like Protein Containing CARD
BD: Becton Dickinson
DPBS: Dulbecco's phosphate-buffered saline
F1: Generation 1 offspring
F1 LPS: Generation 1 offspring, from LPS-injected mothers
F2: Generation 2 offspring
F2 LPS: Generation 2 offspring, from parents born to LPS-injected mothers
FSC: Forward scatter
GC: Glucocorticoid
GR: Glucocorticoid receptor
HPA: Hypothalamic-pituitary-adrenal axis
IL-1: Interleukin-1
IL-6: Interleukin-6
IL-10: Interleukin-10
IFN-*γ*: Interferon-gamma
iNKT: Invariant natural killer T-lymphocyte
MFI: Median fluorescent intensity
MPSI: Maternal periconception systemic inflammation
NK: Natural killer cell
NKT: Natural killer T-(lymphocyte)
NLRP3: Nucleotide-binding domain and leucine-rich repeat containing protein 3
RPMI 1640: Roswell Park Memorial Institute 1640
FSC: Forward scatter
SCC: Side scatter
T_H_1: Type I helper T-lymphocyte
T_H_2: Type II helper T-lymphocyte
TLR4: Toll-like receptor 4
TNF-*α*: Tumour necrosis factor alpha
WBC: White blood cell count.

## Supplementary Materials

Supplementary Materials Supplementary Material I: comparative data on breeding, mating partner, and offspring size across generations. Supplementary Material II: the basal corticosterone levels for F0 mothers, their F1 offspring, and subsequent F2 offspring in plasma. Supplementary Material III: in vitro basal and acute LPS-induced cytokine release by splenocytes of mothers (F0) at the time of sample collection, which took place 4 weeks after the last LPS injection. Supplementary Material IV: in vitro basal cytokine release by splenocytes isolated from F1 and F2 offspring.
