# Supplementary material for: Chronic Gestational Inflammation: Transfer of Maternal Adaptation over Two Generations of Progeny
Source: Mediators Inflamm. 2019 Aug 25;2019:9160941. doi: 10.1155/2019/9160941 (PMC6754931; doi:10.1155/2019/9160941)
Supplement: Supplementary Materials — Supplementary Material I: comparative data on breeding, mating partner, and offspring size across generations. Supplementary Material II: the basal corticosterone levels for F0 mothers, their F1 offspring, and subsequent F2 offspring in plasma. Supplementary Material III: in vitro basal and acute LPS-induced cytokine release by splenocytes of mothers (F0) at the time of sample collection, which took place 4 weeks after the last LPS injection. Supplementary Material IV: in vitro basal cytokine release by splenocytes isolated from F1 and F2 offspring. [file 9160941.f1.docx]

**Supplementary Material I: Breeding data, offspring litter size, gestational length**

| Breeding | Treatment​ | **N** | Mating Partner​ | Gestation days | Litter size Mean ± SEM​ |
| --- | --- | --- | --- | --- | --- |
| F0 ​ | Saline​ | 5 | Wild-type​ | 20 | 6,5 ± 1,26 |
| F0 ​ | LPS​ | 6 | Wild-type​ | 20 | 6,0 ± 0,95 |
| F1​ | Saline​ | 8 | Wild-type​ | 20 | 5,25 ± 0,48 |
| F1 | LPS​ | 8 | Wild-type​ | 20 | 5,83 ± 0,88 |
| LPS; Lipopolysaccharide | | | | | |

**Supplemental material II: Plasma corticosterone levels for F0, F1 and F2 generations**

**Figure 1** Basal plasma corticosterone for LPS-exposed and control groups for F0 generation 4 weeks after weaning of offspring. Data is represented mean ±SEM, F0 Control, n=5; F0 LPS, n=6.

**Figure 2** Basal plasma corticosterone for LPS-exposed and control groups for F1 generation. Data is represented mean ±SEM, F1 Control, n=6; F1 LPS, n=6.

**Figure 3** Basal plasma corticosterone for LPS-exposed and control groups for F2 generation. Data is represented mean ±SEM, F2 Control, n=8; F2 LPS, n=8.

**Supplemental Material III: Ex Vivo Cytokine Response**

**Figure 1** Basal ex vivo cytokine responses of splenic leukocytes from control vs LPS-affected mice for the F0 generation. IFN-γ (a), IL-1β (b), IL-6 (c), TNF-α (d) IL-10 (e) levels were analysed after 18-hour incubation with RPMI 1640. Data is depicted as mean ± SEM, n = 5 and n = 6 for F0 Control and F0 LPS respectively.

**Figure 2** LPS-stimulated ex vivo cytokine responses of splenic leukocytes from control vs LPS-affected mice for the F0 generation. IFN-γ (a), IL-1β (b), IL-6 (c), TNF-α (d) IL-10 (e) levels were analysed after 18-hour incubation with 1ug/ml LPS. Data is depicted as mean ± SEM, n = 5 and n = 6 for F0 Control and F0 LPS respectively.

**Supplemental Material IV: F1 and F2 *Ex Vivo* Cytokine Response**

**Figure 1** Basal ex vivo cytokine responses of splenic leukocytes from control vs LPS-affected mice for the F1 and F2 generation. IFN-γ (a), IL-1β (b), IL-6 (c), TNF-α (d) IL-10 (e) levels were analysed after 18-hour incubation with RPMI 1640. Data is depicted as mean ± SEM, n = 5 and n = 6 for F0 Control and F0 LPS respectively.
